# A Genetically Encoded FRET Lactate Sensor and Its Use To Detect the Warburg Effect in Single Cancer Cells

**DOI:** 10.1371/journal.pone.0057712

**Published:** 2013-02-26

**Authors:** Alejandro San Martín, Sebastián Ceballo, Iván Ruminot, Rodrigo Lerchundi, Wolf B. Frommer, Luis Felipe Barros

**Affiliations:** 1 Centro de Estudios Científicos, Valdivia, Chile; 2 Universidad Austral de Chile, Valdivia, Chile; 3 Carnegie Institution for Science, Stanford, California, United States of America; University of Mississippi, United States

## Abstract

Lactate is shuttled between and inside cells, playing metabolic and signaling roles in healthy tissues. Lactate is also a harbinger of altered metabolism and participates in the pathogenesis of inflammation, hypoxia/ischemia, neurodegeneration and cancer. Many tumor cells show high rates of lactate production in the presence of oxygen, a phenomenon known as the Warburg effect, which has diagnostic and possibly therapeutic implications. In this article we introduce Laconic, a genetically-encoded Forster Resonance Energy Transfer (FRET)-based lactate sensor designed on the bacterial transcription factor LldR. Laconic quantified lactate from 1 µM to 10 mM and was not affected by glucose, pyruvate, acetate, betahydroxybutyrate, glutamate, citrate, α-ketoglutarate, succinate, malate or oxalacetate at concentrations found in mammalian cytosol. Expressed in astrocytes, HEK cells and T98G glioma cells, the sensor allowed dynamic estimation of lactate levels in single cells. Used in combination with a blocker of the monocarboxylate transporter MCT, the sensor was capable of discriminating whether a cell is a net lactate producer or a net lactate consumer. Application of the MCT-block protocol showed that the basal rate of lactate production is 3–5 fold higher in T98G glioma cells than in normal astrocytes. In contrast, the rate of lactate accumulation in response to mitochondrial inhibition with sodium azide was 10 times lower in glioma than in astrocytes, consistent with defective tumor metabolism. A ratio between the rate of lactate production and the rate of azide-induced lactate accumulation, which can be estimated reversibly and in single cells, was identified as a highly sensitive parameter of the Warburg effect, with values of 4.1 ± 0.5 for T98G glioma cells and 0.07 ± 0.007 for astrocytes. In summary, this article describes a genetically-encoded sensor for lactate and its use to measure lactate concentration, lactate flux, and the Warburg effect in single mammalian cells.

## Introduction

Lactate is an organic anion that participates in the intermediate metabolism of eukaryotic and prokaryotic cells. In mammalian cells, lactate is produced from pyruvate by the cytosolic enzyme lactate dehydrogenase (LDH) and is exchanged with the interstitial space and between subcellular compartments via monocarboxylate transporters (MCTs). Hypoxic tissues and tumors release large amounts of lactate, and it was once thought that lactate release was always pathological, but it is now becoming apparent that in addition to its role in hypoxia, lactate has important functions in healthy oxygenated tissues. Intercellular and subcellular exchanges of lactate, termed lactate shuttles, are an integral part of the normal energy metabolism of muscle and brain [1], [2]. In brain tissue, despite normal or elevated oxygen tissue levels, neural activity is accompanied by an acute rise in tissue lactate. Whether and when neurons produce or consume lactate during neural activity remains a controversial issue [3]–[7], which would greatly benefit from lactate measurements in individual cells. In addition, lactate supports the myelination process [8], can behave as an intercellular signal in neurovascular coupling and sodium sensing [9], [10], controls its own production [11] and is required for long-term memory formation [12], [13]. Pathophysiological roles for lactate include inflammation, wound healing, microbial infection, neurodegeneration and cancer [14]–[18].

Standard methods to measure lactate are based on enzymatic reactions that are followed by photometric or amperometric procedures. These methods are limited as they need to consume substrate and/or require destruction of the sample; none of them is capable of detecting intracellular lactate non-invasively in real-time or with single cell resolution. The present article describes a genetically-encoded reporter for lactate, use of this reporter for the determination of lactate transport and metabolic flux with improved spatiotemporal resolution, and the design of a sensitive parameter of cancer metabolism.

## Results

### LldR Flanked by the FRET Pair mTFP-Venus Reports [Lactate]

**Figure 1:**
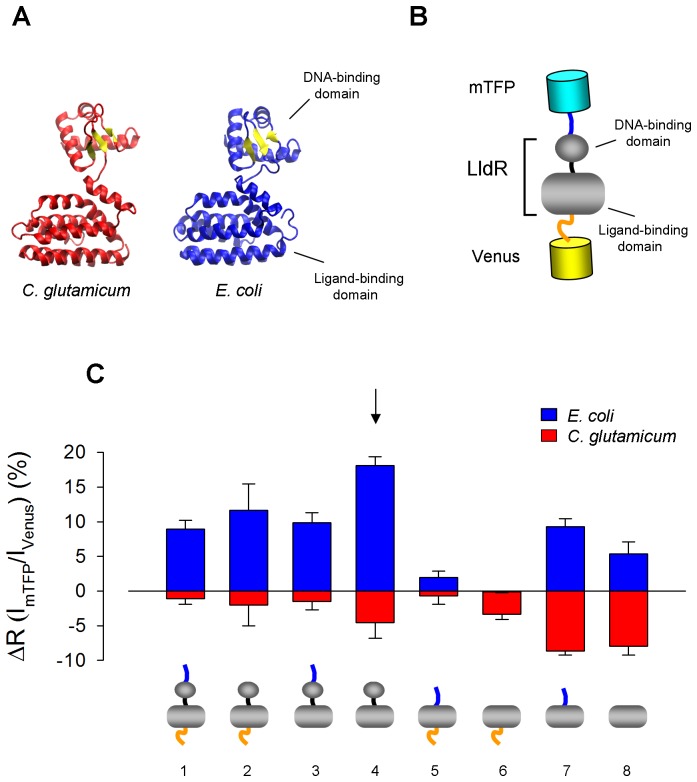
Laconic, a FRET lactate sensor based on the transcriptional regulator LldR. (A) Crystallographic structure of LldR from *Corynebacterium glutamicum* [19], and 3D-structure of LldR from *Escherichia coli* predicted using LldR from *C. glutamicum* and FadR from *E. coli* as templates (M4T Server 3.0 from the Fiser Laboratory http://www.fiserlab.org/servers_table.htm). (B) General design: the transcriptional regulator LldR is sandwiched between fluorescent proteins mTFP and Venus, with artificial peptides separating the proteins (blue and orange linkers). (C) Effect of 10 mM lactate on the fluorescence ratio of 8 variants of the lactate sensor based on LldR from either *E. coli* or *C. glutamicum*. The most responsive of the constructs, indicated by the arrow, was used in the rest of the study.

**Figure 2:**
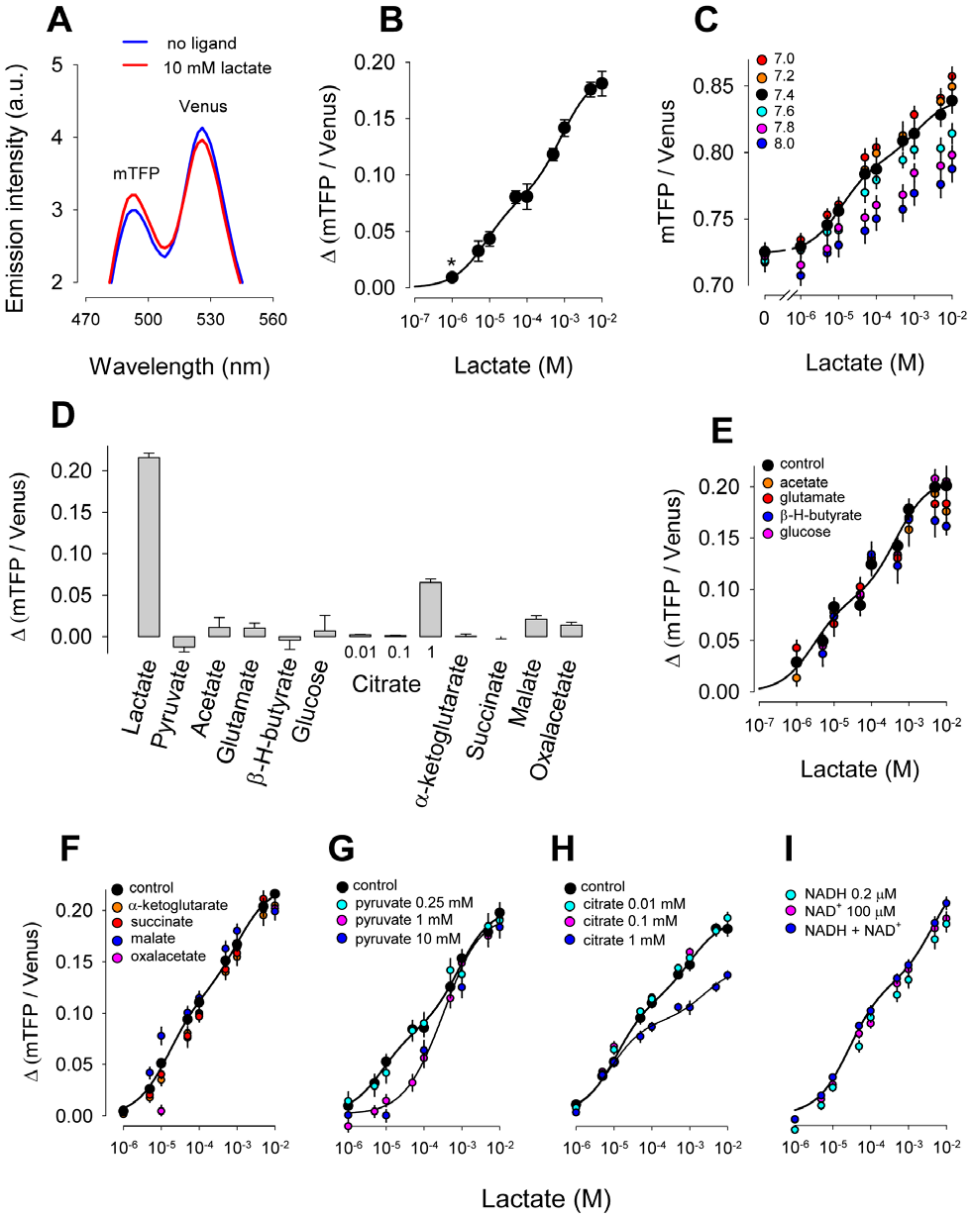
*In vitro* characterization of Laconic. (A) Emission spectra in the absence and presence of 10 mM lactate. (B) The ratio between mTFP and Venus fluorescence (at 430 nm excitation) was measured at 0, 0.001, 0.005, 0.01, 0.05, 0.1, 0.5, 1, 5 and 10 mM lactate. The continuous line corresponds to the best fit of a double rectangular hyperbola to the data, with apparent dissociation constant (K_D_) values of 8 ± 2 µM and 830 ± 160 µM, and respective maximum ΔR values of 8 ± 0.4% and 11 ± 0.4%. (C) Lactate dose-response curves were measured at the indicated pH values. The continuous line corresponds to the best fit of a double rectangular hyperbola to the data at pH .4. (D) Response of the sensor to 5 mM of lactate, pyruvate, acetate, glutamate, β-hydroxy-butyrate and glucose, 1 mM of α-ketoglutarate, succinate, malate or oxalacetate, or increasing concentrations of citrate (0.01, 0.1 and 1 mM). In panels E to I, lactate dose-response curves were measured in the presence of 1 mM acetate, glutamate, β-hydroxy-butyrate or glucose (E), in 1 mM of α-ketoglutarate, succinate, malate or oxalacetate (F), in 0.25, 1 mM or 10 mM pyruvate (G), in 0.01, 0.1 mM or 1 mM citrate (H), and in 0.2 µM NADH, 100 µM NAD+, or 0.2 µM NADH plus 100 µM NAD+ (I). The continuous lines correspond to the best fit of a double rectangular hyperbola to control data.

Genetically-encoded Förster Resonance Energy Transfer (FRET) nanosensors have been developed for measuring the dynamic changes in concentration of several molecules of biological interest with improved spatiotemporal resolution. FRET sensors are fusion proteins composed of a ligand-binding moiety, the recognition element, and a fluorescent pair with overlapping emission and excitation spectra, typically CFP and YFP. Binding of the test molecule causes a conformational change that affects the relative distance and/or orientation between the fluorescent proteins, causing an increase or a decrease in FRET efficiency. The nanosensor described here is based on LldR, a bacterial transcription regulator that consists of two modules, a lactate-binding/regulatory domain and a DNA-binding domain [19], [20]. To generate a lactate sensor, we selected LldR genes from *Corynebacterium glutamicum* and from *Escherichia coli* as potential recognition elements. The three-dimensional structure of the two lactate binding proteins is virtually superimposable (Fig. 1A), yet they are only 19.4% identical, differing in numerous charged residues that may alter surface-charge scanning and possibly the change in FRET efficiency [21]. As a FRET pair we selected mTFP [22] and Venus [23], which, when compared with CFP and YFP, are brighter and less pH-sensitive. The general architecture of the sensors is pictured in Fig. 1B, with mTFP located at the N-terminus, the LldR flanked by linkers, and Venus located at the C-terminus. Eight variants were constructed for each of the two genes using site-specific recombination. The variants differ with respect to the presence of DNA-binding domain and the linker length/composition (sequences are available in Fig. S1). A comparative analysis showed that in response to lactate *E. coli* and *C. glutamicum chimeras* changed their fluorescence ratio in opposite direction, and that the constructs from *E. coli* were more responsive to lactate. Surprisingly, the DNA-binding domain was important for the FRET change. As shown previously for glucose nanosensors [21], lactate sensors lacking artificial linkers performed better (Fig. 1C). The variant with the highest absolute ratio change was chosen for further characterization. This nanosensor termed Laconic (LACtate Optical Nano Indicator from CECs) contains the full-length LldR from *E. coli* without artificial linkers. The emission spectrum of Laconic was characterized by the expected peaks of mTFP and Venus fluorescence at 492 nm and 526 nm, respectively, and a decrease in FRET efficiency upon lactate binding (Fig. 2A). The kinetics of LldR for L-lactate are not known. Figure 2B shows that Laconic detected lactate over four orders of magnitude (from 1 µM to 10 mM), instead of the two orders afforded by one-site sensors such as the glucose nanosensors [24]. When analyzed *in vitro*, Laconic is at least as sensitive as the best commercially available enzyme-based kit. Lactate-dose response curves were determined at different pH values (Fig. 2C), showing a small effect at acidic values and a more marked effect at alkaline values. Specificity was investigated by exposing the sensor to a panel of metabolites and some interference was detected for pyruvate and citrate (Figs. 2D-I). Pyruvate did not change the FRET ratio (Fig. 2D) but at high concentrations, pyruvate blocked the effect of low lactate (Fig. 2G). The physiological concentration of pyruvate in mammalian cells is lower than 100 µM and 10–50 times lower than that of lactate [1], and therefore endogenous pyruvate should not interfere with lactate sensing under physiological conditions. Citrate at 1 mM increased the FRET ratio by 6% (Fig. 2D) and reduced the response of the sensor to high lactate (Fig. 2H). No effects were observed at 10 µM or 100 µM citrate (Figs. 2D and 2H). Cytosolic citrate is lower than 100 µM [25]–[27] and is therefore not expected to interfere with lactate sensing in the cytosol. However, mitochondrial sensing may be affected as mitochondrial citrate is 10 times higher. Extreme redox ratios achieved with combinations of NADH and NAD^+^ [28], were without apparent effect on lactate sensing (Fig. 2I).

### Laconic Reports Cytosolic Lactate in Mammalian Cells

**Figure 3:**
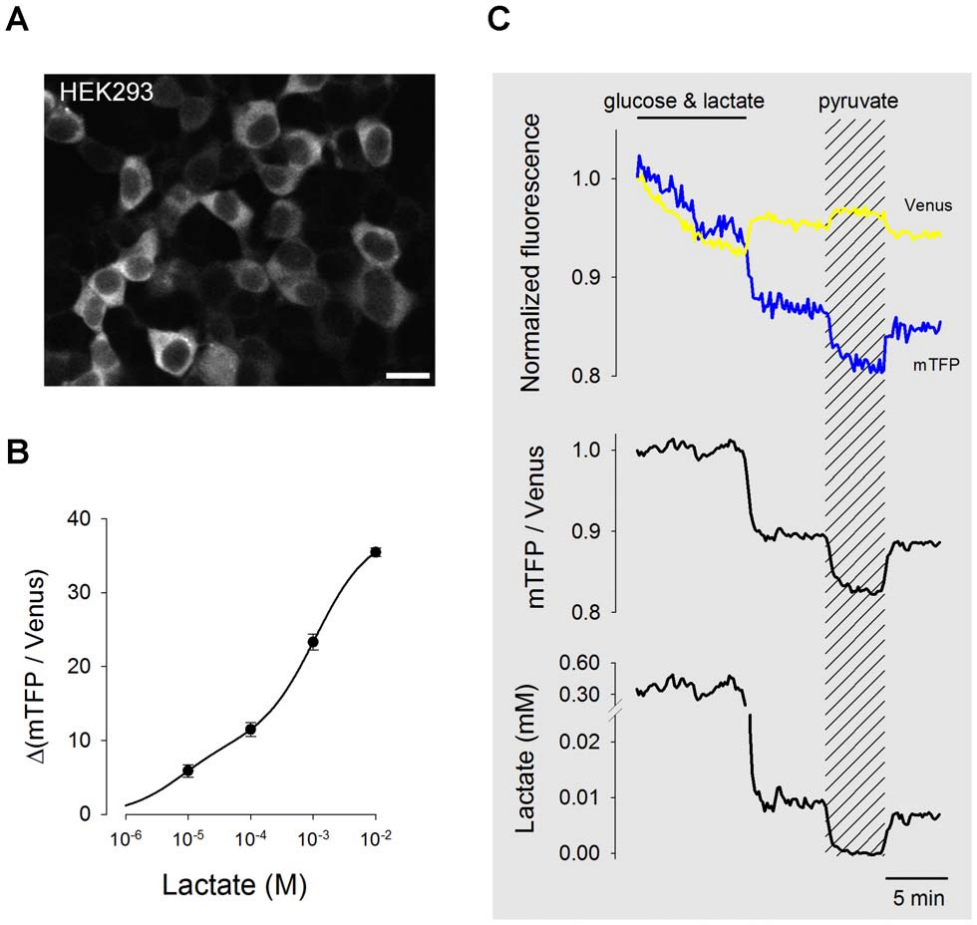
Imaging of cytosolic lactate. (A) HEK293 cells expressing Laconic imaged at 440 excitation/535 emission). Scale bar is 20 µm. (B) Fluorescence ratio was measured at 0.01, 0.1, 1 and 10 mM extracellular lactate in HEK293 cells treated with metabolic inhibitors and permeabilized to H^+^ as described in Material and Methods. (C) The time course of mTFP and Venus fluorescences were measured in the presence of 2 mM glucose/1 mM lactate or 10 mM pyruvate, as indicated. A cell with low expression of the sensor requiring strong illumination was chosen to demonstrate the insensitivity of the fluorescence ratio to photobleaching. The fluorescence ratio was converted into lactate concentration using the ratio in pyruvate (zero lactate) as described in the text.

The sensor displayed the expected cytosolic distribution with nuclear exclusion when expressed in HEK293 cells (Fig. 3A). Although most of the sensor was found in the cytosol, the possibility that a small fraction localizes to the nucleus can not be excluded. However, it does not seem likely that the sensor binds to mammalian DNA, as this type of helix-turn-helix transcriptional regulator is not expressed in mammalian cells. More directly, the binding of LldR to prokariotic DNA requires the 17-nucleotide sequence AATTGGCCCTACCAATT [20], which according to a blast analysis is absent in the mammalian genome. The sequence is also absent in eukaryotic promoters (EPD, Eukaryotic Promoters Database, ExPASy). The possibility of sensor interference with transcription, which we do not favor, may be tackled by substituting Ile 23 with serine, recently reported to eliminate the DNA-binding activity of LldR [29].

For calibration, glycolysis was blocked with iodoacetic acid and mitochondrial metabolism was blocked with rotenone, in the absence of glucose, and the cell was pH-clamped with the proton ionophore nigericine in a potassium-rich buffer. With lactate flux halted and no pH gradient, the MCT was used to equilibrate lactate across the plasma membrane. The resulting calibration curve (Fig. 3B) was similar in shape to that observed with purified protein, suggesting that the sensor *in situ* behaves like *in vitro*. However, the change in mTFP/Venus ratio at all lactate concentrations was about twice that observed *in vitro*. We do not know why the dynamic range of the sensor is higher in cells. Possible explanations include negative interference by the histidine tag used for *in vitro* purification, and/or better stabilization of the protein in the intracellular milieu. The sensor could not be calibrated in astrocytes because in these cells glucose deprivation failed to deplete lactate, as shown by a robust response to pyruvate (see below) and lack of response to low lactate concentrations (data not shown), possibly explained by sustained lactate production from glycogen and/or different MCT affinity. Even in HEK cells, MCT permeability is not linear and the severe cell damage inflicted by the calibration procedure may have altered the sensor protein. For this reason, a less invasive single-point calibration protocol was devised that can be applied in the course of experiments without damaging cells. To deplete cells of lactate, we took advantage of a property of MCTs and other facilitative transporters termed trans-acceleration or accelerated exchange [30]. Thermodynamic equilibrium for the MCT depends on the gradients of lactate and pH but is also affected by the gradient of any other substrate. A forced inward pyruvate gradient was applied to increase the number of inward-facing empty binding sites and decrease the number of outward-facing empty binding sites, increasing lactate efflux and decreasing lactate influx. A new thermodynamic equilibrium may only be reached when the intra:extracellular lactate ratio equals the intra:extracelullar pyruvate ratio. As extracellular lactate was kept close to zero by superfusion of a lactate-free solution, intracellular lactate is predicted to fall, as observed in Fig. 3C. Monochloracetate (MCA), an MCT substrate reduced the fluorescence ratio to a similar extent as pyruvate (Fig. S2), indicating that lactate production from pyruvate is negligible compared with lactate extrusion through the MCT. This can be explained by the fact that the combination of high pyruvate and no glucose severely depletes NADH [28], [31], limiting the LDH reaction. Measurement with an enzymatic kit confirmed that MCA reduces astrocytic lactate (Fig. S2). With the value for the fluorescence ratio at ‘zero’ lactate, the kinetic constants determined *in vitro*, and assuming a maximum change of fluorescence ratio of 38% (from the dose-response curve fitted to HEK cell data), fluorescence data were converted into lactate concentrations as illustrated in Fig. 3C. Further validation of the zero lactate protocol was provided by the observation that after prolonged glucose/lactate deprivation in HEK cells, pyruvate did not affect the fluorescence ratio (data not shown), consistent with full lactate depletion and a very low NADH:NAD^+^ ratio present in glucose-deprived cells [28], [31]. The above evidence plus the *in vitro* observation that the sensor does not respond to low lactate in the presence of high pyruvate (Fig. 2G), suggests that the fluorescence ratio in the presence of high pyruvate is a good estimate of zero lactate. Still, even a small residual signal may introduce a significant bias and caution is advised when using the sensor to quantify lactate, particularly in astrocytes.

By controlling the exchange of lactate between cells and the interstitial space, MCTs are nodal points of tissue metabolism. The uptake of 5 mM lactate by astrocytes was inhibited by 65 ± 3% and 62 ± 7% in the presence of the MCT blockers phloretin and parachloromercurybenzoate (pCMBS; bar graph in Fig. S3). Transformation of lactate to pyruvate by LDH will decrease the rate of lactate accumulation, but because pyruvate concentrations are one order of magnitude lower than those of lactate, this effect should be small. We do not expect lactate buffering by LDH or by the sensor itself to be a major factor because intracellular lactate, typically in range of hundreds of micromolar to millimolar, is more abundant than either [32], [33].

### Measurement of Lactate Production and Consumption

**Figure 4:**
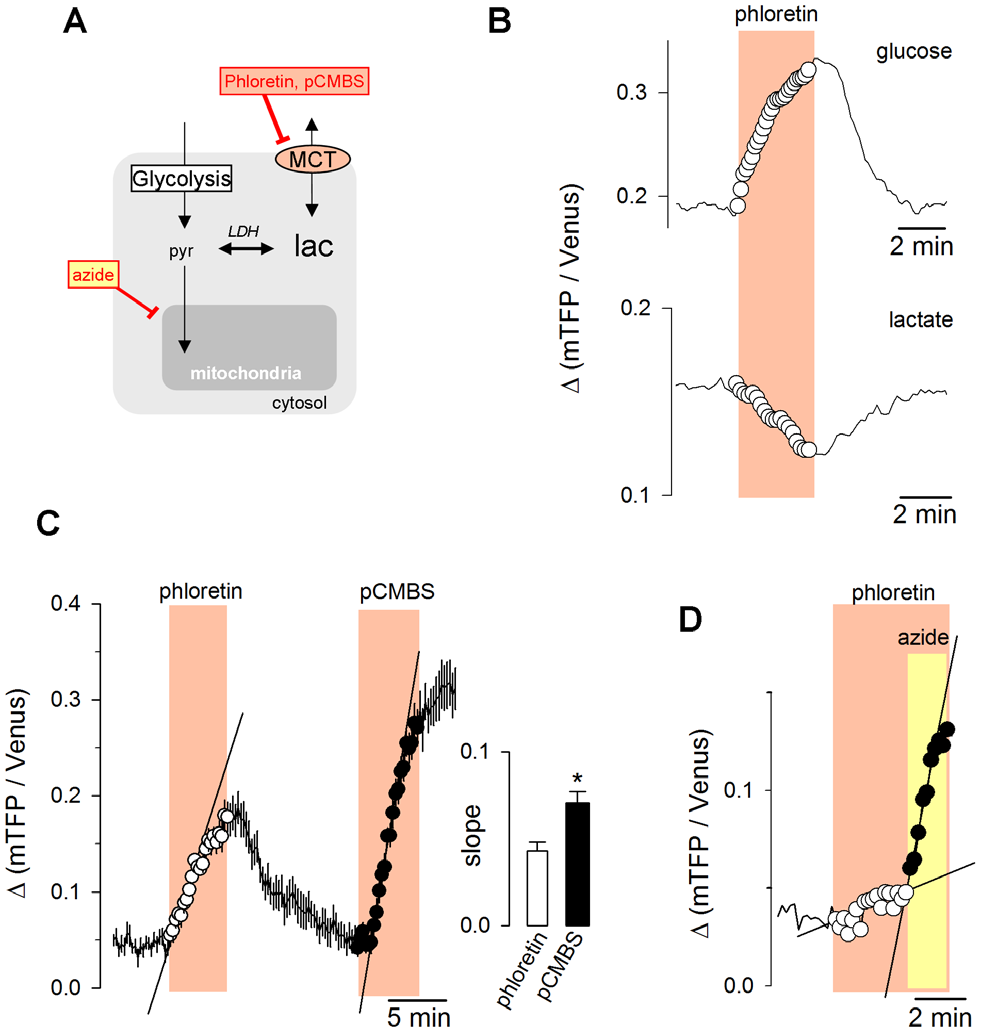
Determination of metabolic fluxes. (A) Diagram of lactate metabolism. The cytosolic concentration of lactate depends on the balance between glycolytic production, mitochondrial consumption of pyruvate and lactate, and the exchange with the extracellular lactate pool via MCTs. (B) Intracellular lactate was monitored in individual HEK293 cells during transient exposures to phloretin (50 µM), in the presence of 25 mM glucose (top panel) or 1 mM lactate (bottom panel). (C) The responses of intracellular lactate to transient inhibitions of the MCT with phloretin (50 µM) or pCMBS (500 µM) were sequentially measured in the same HEK293 cell in the presence of 25 mM glucose. The straight lines represent slopes of lactate accumulation fitted by linear regression during the first minute of exposure. The bar graph summarizes data from three experiments. (D) The effect of 5 mM sodium azide was measured in an astrocyte during exposure to 50 µM phloretin.

The flux diagram in Fig. 4A shows how the intracellular concentration of lactate is affected by glycolytic production, mitochondrial consumption and export/import through the MCT. The rate of glycolysis at its entry point has been estimated with a FRET glucose sensor by blocking the glucose transporter GLUT while monitoring the rate at which hexokinase depletes the intracellular pool of glucose [34], a technique that has served to detect fast changes in astrocytic glycolysis in response to neuronal signals [11], [35], [36]. Following a similar principle, lactate exchange was investigated in HEK293 cells by perturbing the steady state with an MCT blocker while measuring cytosolic lactate with Laconic. In the presence of glucose as exclusive fuel, phloretin caused an accumulation of intracellular lactate, indicative of net lactate production, but if glucose was replaced by lactate, the result was lactate depletion (Fig. 4B), indicative of net lactate import. In HEK cells phloretin inhibited only 57±5% of MCT activity (Fig. S3), and therefore underestimates the actual rates of production or consumption by about 40%. pCMBS was a more effective MCT blocker than phloretin in HEK cells, with 96 ± 1% inhibition (Fig. S3), giving a more accurate estimate of lactate production (Fig. 4C). In astrocytes, the rate of lactate production calculated with the calibration procedure described in Fig. 3C was 2±0.5 µM/s (n = 22 cells in three experiments), which falls in the right order of magnitude considering that under identical culture and experimental conditions astrocytes consumed glucose at 2 µM/s [34] and that some glucose is oxidized to CO_2_. Acute inhibition of oxidative phosphorylation (OXPHOS) with azide stimulated lactate production by several fold (Fig. 4D), fitting the observed stimulation of glucose consumption [34]. Control pH measurements with BCECF showed that azide caused a small acidification that in astrocytes reached 0.1 pH units over the period in which lactate accumulation was determined (Fig. S4). By 2 min of azide exposure, the three cell types had stabilized at a pH about 0.2 pH units lower than control values. The effects of phloretin and pCMBS on intracellular pH were smaller. Considering the modest sensitivity of the lactate sensor to a small acidification (Fig. 2C), pH does not seem to be a significant factor in the differential effects of the inhibitors to lactate sensing in astrocytes, HEK293 and T98G cells.

### Single-Cell Real-Time Detection of Cancer Metabolism

**Figure 5:**
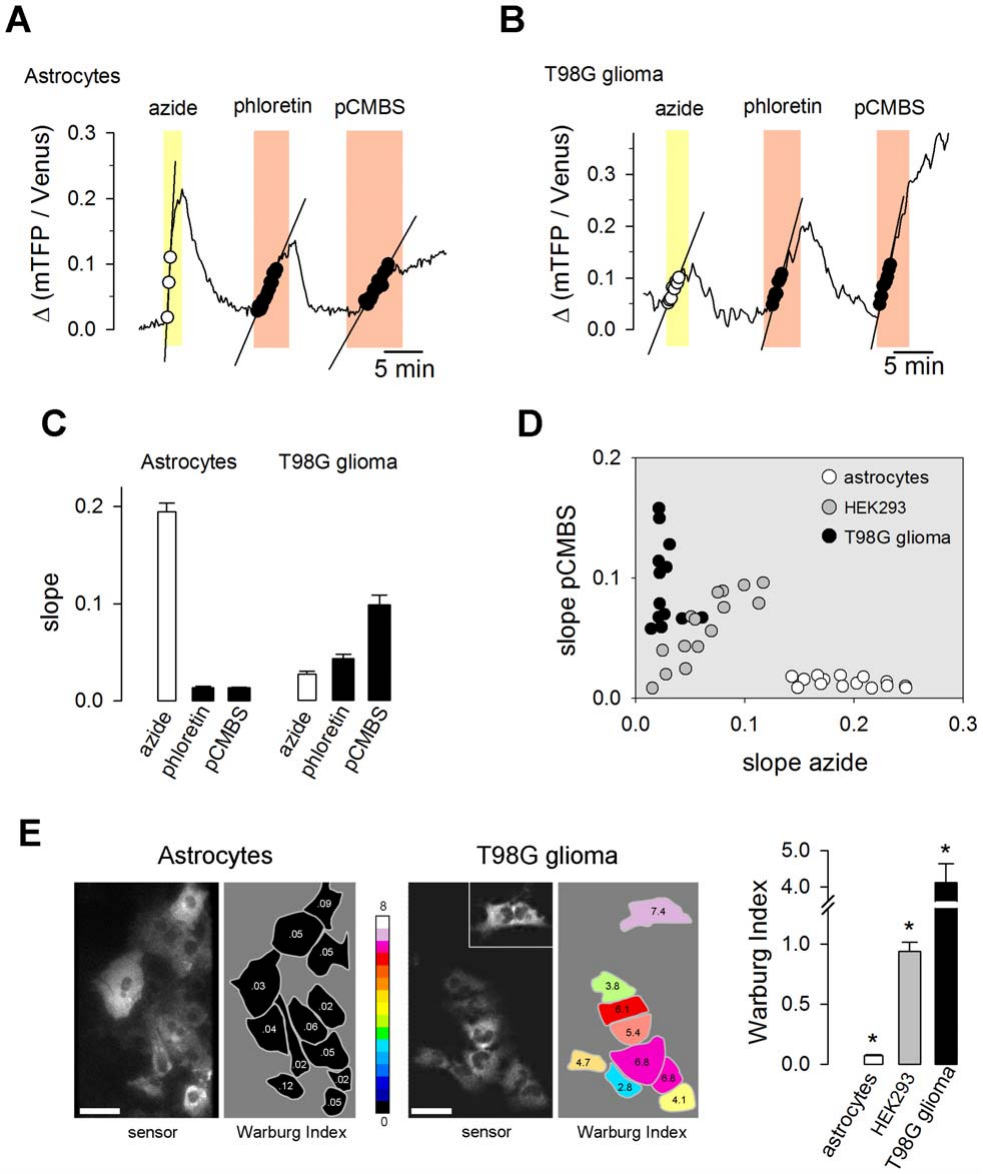
Metabolic characterization of tumor cells. (A) An astrocyte expressing Laconic was sequentially exposed to 5 mM azide, 50 µM phloretin and 500 µM pCMBS. The straight lines represent initial slopes of lactate accumulation fitted by linear regression within the same range of ratio values. (B) A T98G glioma cell expressing Laconic was sequentially exposed to 5 mM azide, 50 µM phloretin and 500 µM pCMBS. The straight lines represent initial slopes of lactate accumulation fitted by linear regression within the same range of ratio values. (C) Summary of the initial slopes (Δ ratio/min) obtained in three experiments of the type shown in A and B. (D) Correlation plot between the rates of lactate accumulation (Δ ratio/min) in azide and in pCMBS. Symbols represent single astrocytes (white), HEK293 cells (gray) or T98G cells (black). (E) The Warburg Index was estimated as the ratio between the rates of lactate production with pCMBS and lactate accumulation with azide, and used to color the silhouette of each cell according to the 16-color look up table. The inset shows an isolated cell that was located about 100 µm from the cluster. The bar graph summarizes data from 3 experiments in each cell type. Scale bars are 20 µm. *, p < 0.05 between every cell type.

Cancer cells have defective mitochondria and strong glycolysis, a phenomenon known as the Warburg effect, which is also present in cell lines [15]. Consistent with a minor role for mitochondria in ATP production in cancer cells, real-time glucose measurement with the FRET glucose sensor [37] showed that glycolysis in T98G glioma cells is much less responsive to OXPHOS inhibition than glycolysis in astrocytes (Fig. S5). Thus, a robust glycolytic response to azide can be interpreted as a sign that mitochondria have a significant role in cellular ATP production. The reversibility of the effects of azide and phloretin allowed sequential applications of azide, phloretin and pCMBS to the same cell. The results show astrocytes and glioma cells presenting opposite patterns, with astrocytes responding strongly to azide but weakly to phloretin/pCMBS and T98G cells responding weakly to azide and more strongly to phloretin/pCMBS (Figs. 5A-D). An analysis of the frequency of acquisition required for accurate estimation of the response to azide is described in Fig. S6. Control experiments showed that as observed in HEK cells, in T98G glioma cells pCMBS is a better inhibitor of the MCT than phloretin (95 ± 5% and 53 ± 3% inhibition, respectively; Fig. S3). A ratio between basal lactate production and the response to OXPHOS inhibition measured at the same range of fluorescence ratio and in the same cell should be insensitive to lactate concentration and therefore less affected by possible differences in resting lactate concentration or sensor behavior between cell types. Such a ratio, which we have termed the Warburg Index, was highly sensitive to the metabolic difference between astrocytes and glioma cells. Calculated with phloretin, which underestimates lactate production more in glioma cells and HEK cells than in astrocytes (Fig. S3), the Warburg Index was 0.07 ± 0.01 in astrocytes, 0.74 ± 0.15 in HEK cells and 1.7 ± 0.2 in glioma cells. Calculated with pCMBS, the Warburg Index was 0.07 ± 0.01 in astrocytes, 0.94 ± 0.08 in HEK cells and 4.1 ± 0.6 in glioma cells (Fig. 5E). More specific MCT inhibitors are being introduced in research and in clinical trials that can be used at micromolar concentrations or lower. AR-C155858, a specific inhibitor of MCT1 and MCT2 [38], was found to inhibit the uptake of 5 mM lactate in astrocytes by 87% (Fig. S7). Using AR-C155858 the Warburg Index estimated in astrocytes was 0.07 ± 0.006 (Fig. S7). Control experiments showed that phloretin, azide and AR-C155858 were without effect on lactate sensing *in vitro* (Fig. S8). pCMBS is not expected to interact with the sensor as it does not enter mammalian cells.

## Discussion

We have used the bacterial transcriptional regulator LldR as recognition element to generate Laconic, a genetically-encoded biosensor that detects lactate in the physiological range. Expressed in mammalian cells, the sensor was capable of sensitive estimation of lactate transporter activity and lactate production and was used to generate a novel single-cell parameter of the Warburg effect, the metabolic trademark of cancer cells. The development of a sensor based on LldR provides the basis for creating a wide variety of novel indicators because the GntR superfamily, of which LldR is a member, comprises at least 270 other transcription factors that bind pyruvate, fatty acids, amino acids, TCA cycle intermediates, etc. [19], [39], which are possible recognition elements for genetically-encoded nanosensors.

The sensor was capable of quantifying lactate levels in the range between 1 µM and 10 mM. The extended range of detection was unexpected as previously developed metabolite sensors span only two orders of magnitude [40]–[43]. Affinity is chiefly determined by the properties of the recognition element and not by the fluorescent partners, so the distinctive kinetic behavior of this LldR-based sensor and its sensitivity to mM levels of pyruvate may be of interest for researchers working on transcriptional regulation. Laconic offers advantages over alternative methods, the first being resolution. Enzymatic assays and HPLC demand a large number of cells, rendering meaningful data only if there is metabolic homogeneity. Real tissues and even primary tissue cultures are complex mixtures of cells that grow and differentiate best in the presence of each other, a popular example being co-cultures of neurons and astrocytes. As with other genetically-encoded nanosensors, Laconic may be expressed *in vivo* in identified cell types using transgenesis or viral transduction and may be targeted to subcellular organelles. Another plus of optical measurements is that they do not require destruction of the sample, allowing the design of statistically powerful before-and-after type experiments. In addition to being transported by MCTs, whose activity can be estimated using a pH sensitive dye, lactate is also transported independently of protons through gap junctions [44] and possibly through connexin hemichannels and pannexin channels, fluxes that are invisible to pH measurements and that may now be approached.

Although the similar shape of the calibration curves obtained *in vitro* and in HEK cells is encouraging, the necessary harshness of the full calibration protocol is a matter of concern and it is not possible at this stage to ensure that the kinetic parameters obtained *in vitro* represent the behavior of the sensor in cells. The data may therefore be considered as semi-quantitative and expressed in terms of Δ ratio using the zero lactate point as a reference, ideally in before-and-after experiments with rates compared at similar ratio values. Another limitation of Laconic is its pH sensitivity in the alkaline range. Under physiological conditions the cytosolic pH of most cells fluctuates between 7.0 and 7.4, range in which the sensor showed little change, but given the wide range of concentrations covered, even a relatively small change in pH may introduce an error in lactate determination. If a strong pH change is present, a correction may be accomplished with red pH sensitive proteins or dyes, co-loaded in the same cells or with parallel BCECF experiments. A standing issue in cell metabolism is the extent to which mitochondria metabolize lactate instead of pyruvate [45]–[47], a phenomenon that may be addressed with the lactate sensor expressed in the cytosol. Lactate measurements in the mitochondrial matrix may prove more difficult due to the reduced sensitivity of Laconic to the pH 7.8-8.0 environment of this compartment and possible interference by citrate. These limitations may perhaps be overcome by mutagenesis, as demonstrated for the pH sensitivity of the NADH sensor Peredox [28].

The protocol to assess lactate production/consumption should ideally use a reversible inhibitor capable of blocking lactate permeability without affecting other membrane proteins or intracellular processes. Neither pCMBS nor phloretin fulfill such description, but they complement each other. pCMBS does not enter the cell but targets numerous surface proteins and its effect is irreversible, precluding the design of before-and after experiments. We do not know why astrocytes were more resistant to pCMBS. Differential sensitivity of MCT isoforms is possible, but we could not find evidence of that in the literature. The partial inhibition by pCMBS is expected to underestimate lactate production in astrocytes by about 35%, with a similar bias introduced in the Warburg index. The effect of phloretin is reversible, but less effective and is not specific either. In addition to inhibiting MCTs, both phloretin and pCMBS target the glucose transporter GLUT, but this should not compromise the analysis of astrocytes, neurons, cancer cells, fibroblasts, cell lines in general and other cells that maintain resting intracellular glucose at levels > 300 µM [34], [37], [40]. In such cells, glucose is well above the K_m_ of hexokinase and glucose consumption remains constant for several minutes in the presence of a GLUT blocker [34]. High-affinity MCT blockers have recently been developed by pharmaceutical companies that may be used for the present purpose, when made commercially available. For this article, we tested AR-C155858, a specific inhibitor of MCT 1 and MCT2 [38]. When used with cultured astrocytes and glioma cells AR-C155858 behaved similarly to phloretin and pCMBS (Fig. S6). However, AR-C155858 does not inhibit MCT4, an isoform important in mature astrocytes, muscle cells and some cancer cell lines [48], [49]. The effectiveness of the inhibitor to be selected should be verified with an uptake asssay prior to measuring lactate production. Combined with MCT knockdowns using siRNA or shRNA, the lactate sensor may also be used to discriminate the contribution of specific isoforms to flux of lactate.

The stimulation of glycolysis by azide is explained by cessation of mitochondrial ATP production, possibly exacerbated by additional ATP consumption by the mitochondrial ATP synthase acting in reverse. ATP synthase reversal will exacerbate the glycolytic stimulation by further lowering the ATP/ADP ratio. Addition of the ATP synthase inhibitor oligomycin to azide might provide a more accurate assessment of the stimulation of glycolysis, given the endogenous ATP consumption ocurring in the cell prior to mitochondrial inhibition. We do not think that the slower accumulation of lactate in response to azide in tumour cells is explained by greater lactate transport capacity. In the limit, the initial rate of lactate departure from the steady-state after glycolytic stimulation depends only the resting lactate flux and the degree of stimulation. As cancer cells have higher resting fluxes than normal cells, the initial rate of lactate increase in response to a given glycolytic stimulation is expected to be higher. If in practice, the true initial rate can not be resolved, lactate efflux will become a factor, but the greater lactate transport capacity of cancer cells is predicted to cancel out with the commensurate greater lactate flux. A weak glycolytic response to azide may be due to weak mitochondrial function but also to regulatory defects in glycolysis. If a cell type can not increase its glycolytic rate in response to azide, it may be mistaken as a tumor-type phenotype. However, we reckon that may not be a common occurrence as the Pasteur effect appears to be a universal feature of healthy mammalian cells. This is because most cells have an intracellular glucose pool that together with the pool of glycolytic intermediates will sustain lactate production, at least for a while. Muscle cells have conspicuously low steady-state glucose levels but are known to respond to mitochondrial inhibitors with a robust stimulation of glycolysis from glycogen deposits. Incidentally, we have observed that HEK cells increase their production of lactate in response to azide, even in the absence of extracellular glucose (data not shown).

Cancer cells are more glycolytic than normal cells even in the presence of oxygen, a phenomenon first noted by Otto Warburg and which is receiving renewed attention in relation to cancer pathogenesis, diagnosis and possibly treatment [14], [15], [50]. The molecular mechanisms responsible for the Warburg effect are pleiotropic, involving defects and gain-of function modulations of glycolytic and mitochondrial enzymes, differing between types of cancer and even between cell lineages within a tumor. There is a positive correlation between tumor aggression/resistance to chemotherapy and the intensity of the Warburg effect. This phenomenon is currently detected by comparing the rates of glucose and oxygen consumption or, alternatively, the rates of lactate production and oxygen consumption, estimated in cell populations. The present sensor and its associated protocols contribute to the field by allowing estimation of the Warburg effect in single cells, a useful feature for the study of heterogeneous cell populations.

## Materials and Methods

Standard reagents and inhibitors were acquired from Sigma. AR-C155858 was purchased from Haoyuan Chemexpress (Shanghai)

### Construction of the Lactate Sensor

Bacterial genomic DNA was isolated using Purification System Wizard® SV Genomic DNA (Promega) and LldR genes were amplified with specific primers. To simplify the cloning strategy in *C. glutamicum*, a BamH I site was removed by introducing a single-nucleotide silent mutation. For easy subcloning of LldR sequences, restriction sites AflII and KpnI were respectively introduced at the 3' and 5' end of the amplicons. PCR reactions were carried out with a KOD hot start DNA polymerase (Novagen) and their products were checked by sequencing (Macrogen. Inc). Sixteen variants of the lactate sensor, eight for *E. coli* and eight for *C. glutamicum*, were generated using the Gateway® recombination cloning technology (Invitrogen), according to the manufactureŕs instructions. Briefly, five plasmid vectors were constructed: a destination vector and four entry vectors. The destination vector (pDEST01) comprised the backbone vector pRSET-B in which the DNA sequences coding for a polyhistidine tail, mTFP and Venus were cloned downstream of the bacterial T7 promoter. A recombination cassette with introduced AflII and KpnI sites, amplified from pDEST14 by PCR, was intercalated between the sequences for mTFP and Venus. The four entry vectors were constructed using the pCR®8/GW/TOPO TA vector by cloning the sequences corresponding to the full LldR from *E. coli* (pENTLldR01), the full length of LldR from *C. glutamicum* (pENTLldR02), the ligand-binding domain of LldR from *E. coli* (pENTLldR03), and the ligand binding-domain of LldR from *C. glutamicum* (pENTLldR04). Finally, the sixteen expression vectors were generated by LR recombination between the destination vector pDEST01 and each of the four entry vectors, followed by conditional removal of the linker between mTFP and LldR or the linker between LldR and Venus by digestion of the expression vectors with the restriction enzymes Kpn I and AflII.

### Protein Purification

The sixteen variants were transformed into *E. coli* BL21 (DE3). A single colony was inoculated in 100 ml of LB medium with 100 mg/ml ampicillin (without IPTG) and shaken in the dark for 2–3 days. Cells were collected by centrifugation at 5000 rpm (4 °C) for 10 min and disrupted by sonication (Hielscher Ultrasound Technology) in 5 mL of Tris-HCl buffer pH 8.0. A cell-free extract was obtained by centrifugation at 10,000 rpm (4 °C) for 1 hour and filtering of the supernatant (0.45 µm). Proteins were purified using a Nickel resin (His Bin® from Novagen) as recommended by the manufacturer. Eluted proteins were quantified using the Biuret method and stored at −20 °C in 20% glycerol. The variant that showed the largest change in fluorescence ratio, termed Laconic (Lactate Optical Nanosensor from CECs), was cloned into pcDNA3.1(-) for expression in eukaryotic cells using the restriction sites BamH I and Hind III.

### Animals and Cell Cultures

Animals used were mixed F1 male mice (C57BL/6J × CBA/J), kept in an animal room under SPF conditions at a room temperature of 20±2 °C, in a 12/12 h light/dark cycle with free access to food and water. Experiments were approved by the Centro de Estudios Científicos Animal Care and Use Committee. Mixed cortical cultures of neuronal and glial cells (1–3 day-old neonates) were prepared as described [51]. For the present study only astrocytes were measured. HEK293 and T98G glioma cells were acquired from the American Type Culture Collection and cultured at 37 °C in 95% air/5% CO_2_ in DMEM/F12 10% fetal bovine serum. HEK cells and T98G cells were transfected at 60% confluence using Lipofectamine 2000 (Gibco), with efficiencies of > 90% in HEK cells and approx. 20% in T98G cells. Astrocytes were transfected at 60% confluence using Lipofectamine 2000 (Gibco), with approx. 20% of efficiency, or alternatively, exposed to 5 × 10^6^ PFU of Ad Laconic or Ad FLII^12^Pglu600 µδ6 (Serotype 5, custom made by Vector Biolabs), which transduced > 90% of the cells. There was no apparent difference in the behavior of the sensor in astrocytes transfected with plasmid versus astrocytes transduced with the adenoviral vector. Cells were studied after 24–72 h.

### Fluorescence Measurements

Nickel-purified proteins were resuspended at 100 nM in an intracellular buffer containing (mM): 10 NaCl, 130 KCl, 1.25 MgSO_4_ and 10 HEPES, pH 7.0, and measured with a microplate reader analyzer (EnVision, PerkinElmer). The proteins were excited at 430 nm and the intensity of fluorescence emission of mTFP and Venus were recorded at 485 nm and 528 nm, respectively. The ratio between the emissions was used to characterize the sensors. Emission spectra were obtained at 430 nm excitation, with 2 nm windows. Cells were imaged at room temperature (22–25 °C) in a 95% air/5% CO_2_-gassed solution of the following composition (in mM): 112 NaCl, 1.25 CaCl_2_, 1.25 MgSO_4_, 1–2 glucose, 10 HEPES, 24 NaHCO_3_, pH 7.4, with 3 mM KCl (astrocytes) or 5 mM KCl (HEK293 and T98G) using an upright Olympus FV1000 Confocal Microscope equipped with a 20× water immersion objective (N.A. 1.0) and a 440 nm solid-state laser. Alternatively, cells were imaged with an Olympus IX70 or with an Olympus BX51 microscope equipped with a 40× oil-immersion objective (NA 1.3) or with a 20× water-immersion objective (NA 0.95). Microscopes were equipped with CAIRN monochromators (Faversham, UK), and either a Hamamatsu Orca camera controlled by Kinetics software or a Rollera camera controlled with Metafluor software, respectively. For nanosensor ratio measurements, cells were excited at 430 nm for 0.2–0.8 s. Emission was divided with a CAIRN Optosplit, equipped with band pass filters at 480 ± 20 (mTFP) and 535±15 nm (Venus). The ratio between mTFP and Venus was used to estimate lactate. After individual experiments, the fluorescence ratio at zero lactate was determined in the presence of 10 mM pyruvate (see Fig. 3C). Most data are presented as the difference between fluorescence ratio and fluorescence ratio in the presence of pyruvate, Δ(mTFP/Venus). To generate ratio image from 8-bit background-subtracted images and avoid division by zero, an arbitrary offset of value 5 was added to the Venus image (denominator). The pH-sensitive dye BCECF was ester loaded at 0.1 µM for 3–4 min and the signal was calibrated by exposing the cultures to solutions of different pH after permeabilizing the cells with 10 µg/ml nigericin and 20 µg/ml gramicidin in an intracellular buffer. BCECF was sequentially excited at 440 and 490 nm (0.05 s) and imaged at 535 ± 15 nm. To calibrate the lactate sensor in cells, HEK293 cells were pre-incubated for 1 hour with 500 µM iodoacetic to block glycolysis at GAPDH [52]. Extracellular and intracellular lactate were then equilibrated using the endogenous H^+^-linked monocarboxylate transporter MCT in the presence of the H^+^/K^+^ exchanger nigericine (10 µM) and 2 µM rotenone in an intracellular buffer of the following composition (mM): 10 NaCl, 130 KCl, 1.25 MgSO_4_, 10 HEPES pH 7.2. Intracellular glucose was measured with a FRET sensor [37] as detailed in [34]. To estimate intracellular lactate with a different technique, > 80% confluent pure astrocyte cultures grown on 35 mm Petri dishes were washed thrice with ice-cold imaging buffer supplemented with 50 µM phloretin, and then permeabilized with 500 µl of 10 mM HEPES pH 7.4 and 0.1% triton. Lactate in the extract was measured with the Biovision Lactate Assay Kit according to manufactureŕs instructions.

### Statistical Analysis

Time courses without error bars correspond to single cells. Experiments were repeated three to six times, with 6–18 cells per experiment unless otherwise stated. Regression and statistical analyses were carried out with the computer program SigmaPlot (Jandel). Differences in mean values of paired samples were evaluated with the Students t-test. Differences between more than two groups were evaluated with the Kruskal-Wallis One Way Analysis of Variance on Ranks followed by Dunńs test. P values < 0.05 were considered significant and are indicated with an asterisk (*).

## Supporting Information

Figure S1 **Related to** **Fig. 1** **.** Alignment of lactate sensor sequences. Eight variants of the lactate sensor were generated with either LldR from *E. coli* and *C. glutamicum* as described in Experimental Procedures. Variant 04 from *E. Coli* was termed Laconic. Identical amino acid residues are highlighted in yellow.
(DOC)

Figure S2 **Related to** **Fig. 3** **.** Depletion of lactate induced by pyruvate and monochloracetate. (A) Astrocytes were successively exposed to 5 mM lactate, 10 mM pyruvate and 50 mM monochloroacetate (MCA). Time lapse data are from 5 cells in a single experiment. The bar graph summarizes the difference between control ratios and in the presence of pyruvate or MCA for 3 separate experiments. (B) Whole cell lactate was estimated with an enzymatic kit in astrocytic cultures exposed to 2 mM glucose/1 mM lactate (control), after 5 min exposure to 50 mM MCA, or after 5 min exposure to 5 mM lactate. Data are from 3–4 separate determinations. *, p < 0.05 respect to control.
(DOC)

Figure S3 **Related to** **Fig. 4** **.** Inhibition of lactate uptake by phloretin and pCMBS. T98G glioma cells were exposed to 5 mM lactate in the absence and presence of 50 µM phloretin or 500 µM pCMBS. The straight lines represent the initial slopes of lactate uptake. The bar graphs summarize data for 3 experiments in each cell type. *, p < 0.05 with respect to phloretin.
(DOC)

Figure S4 **Related to** **Fig. 4** **.** Effects of azide, phloretin and pCMBS on pH. Intracellular pH was measured with BCECF in astrocytes exposed to 5 mM azide, 50 µM phloretin or 500 µM pCMBS. A control alkalinization with 10 mM ammonium chloride is also shown. Data are from 8 cells in a single experiment. Arrows show the times selected for the bar graphs below, which summarize the fall in intracellular pH for 3 experiments in each cell type. Note that lactate accumulation measurements in **Fig. 5** were done at 30 s for azide, at 2 min for phloretin and at 4 min for pCMBS.
(DOC)

Figure S5 **Related to** **Fig. 5** **.** Intracellular glucose depletion by azide. The effect of 5 mM azide on intracellular glucose was measured with a FRET glucose sensor (Bitner et al., 2010). The bar graph represents the degree of glucose depletion after 2 min of exposure to azide (arrow) in 3 experiments in each cell type. *, p < 0.05 respect to astrocytes and HEK293 cells.
(DOC)

Figure S6 **related to** **Fig. 5** **.** Effect of frequency of acquisition on the estimation of the lactate rise in response to azide. Astrocytes expressing the lactate sensor in the presence of 2 mM glucose were exposed to 5 mM azide while the fluorescence ratio was measured every second (1 Hz). A linear function was fitted to the initial phase of the lactate rise, giving an estimated rate of 0.23 min^−1^ (left panel). When the data were decimated to simulate a frequency of acquisition of 0.1 Hz, the estimated rate was 0.26 min^−1^(right panel). The lower graph shows data for 3 different cells. Similar results were obtained in a separate experiment.
(DOC)

Figure S7 **Related to** **Fig. 5** **.** Estimation of the Warburg Index with AR-C155858. (**A**) The uptake of 5 mM lactate was measured in astrocytes in the absence and presence of 1 µM AR-C155858. Data are from 10 cells in two experiments. (**B**) An astrocyte expressing Laconic was sequentially exposed to 5 mM azide and 1 µM AR-C155858. The straight lines represent the slopes of lactate accumulation fitted by linear regression within the same range of ratio values. The bar graph shows a summary of the slopes (Δ ratio/min) obtained for 10 cells in two experiments. The calculated Warburg index was 0.07 ± 0.006.
(DOC)

Figure S8 **Related to** **Figs. 4** **–** **5** **.** Effects of phloretin, AR-C155858 and azide on lactate sensing *in vitro.* The mTFP/Venus ratio was measured in the stated conditions and expressed relative to the control value in the absence of lactate. Data are from 6 determinations in two different sensor extracts.
(DOC)
