# Supplementary material for: A Genetically Encoded FRET Lactate Sensor and Its Use To Detect the Warburg Effect in Single Cancer Cells
Source: PLoS One. 2013 Feb 26;8(2):e57712. doi: 10.1371/journal.pone.0057712 (PMC3582500; doi:10.1371/journal.pone.0057712)
Supplement: Figure S1 — Related to Fig. 1 . Alignment of lactate sensor sequences. Eight variants of the lactate sensor were generated with either LldR from E. coli and C. glutamicum as described in Experimental Procedures. Variant 04 from E. Coli was termed Laconic. Identical amino acid residues are highlighted in yellow. (DOC) [file pone.0057712.s001.doc]

**Figure S1. Alignment of lactate sensor sequences**


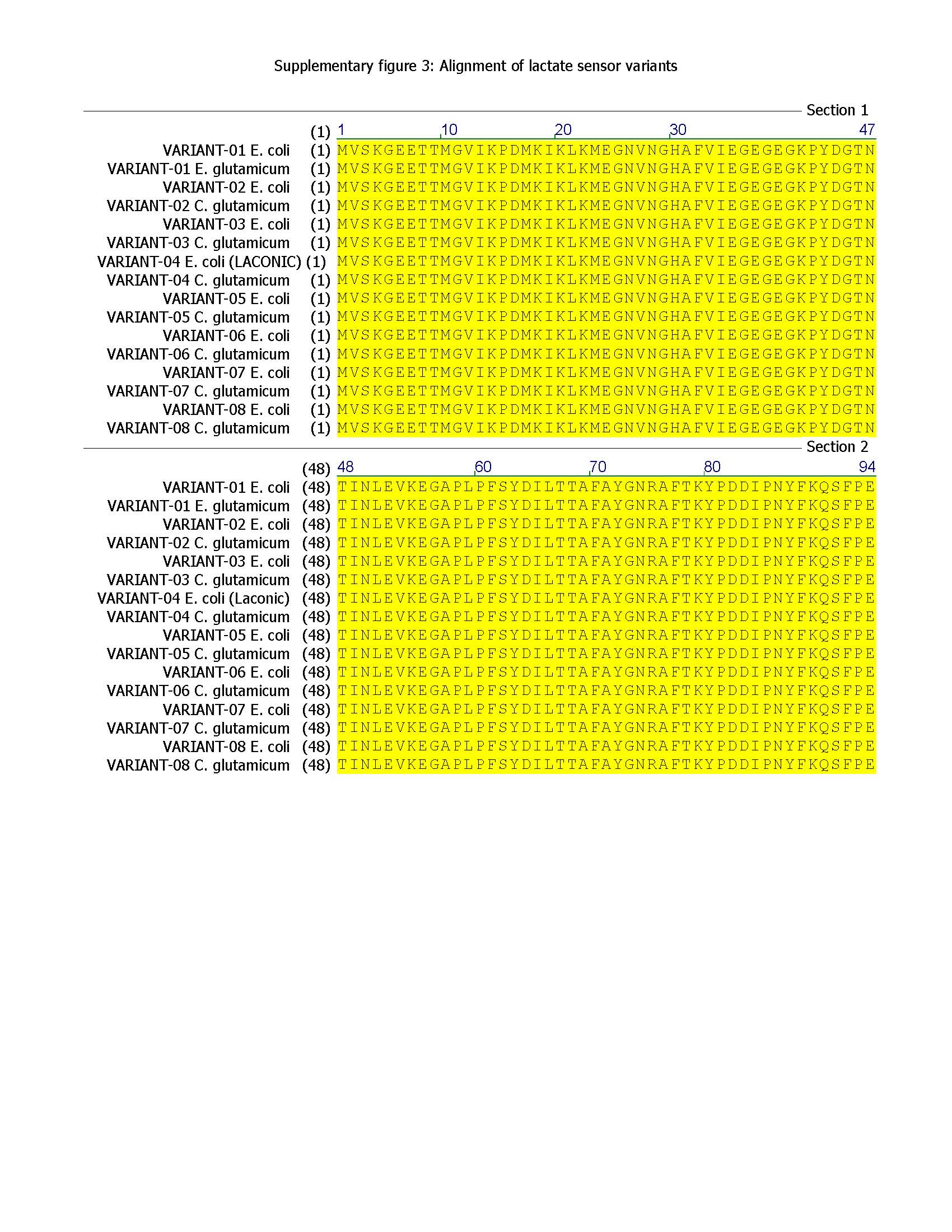

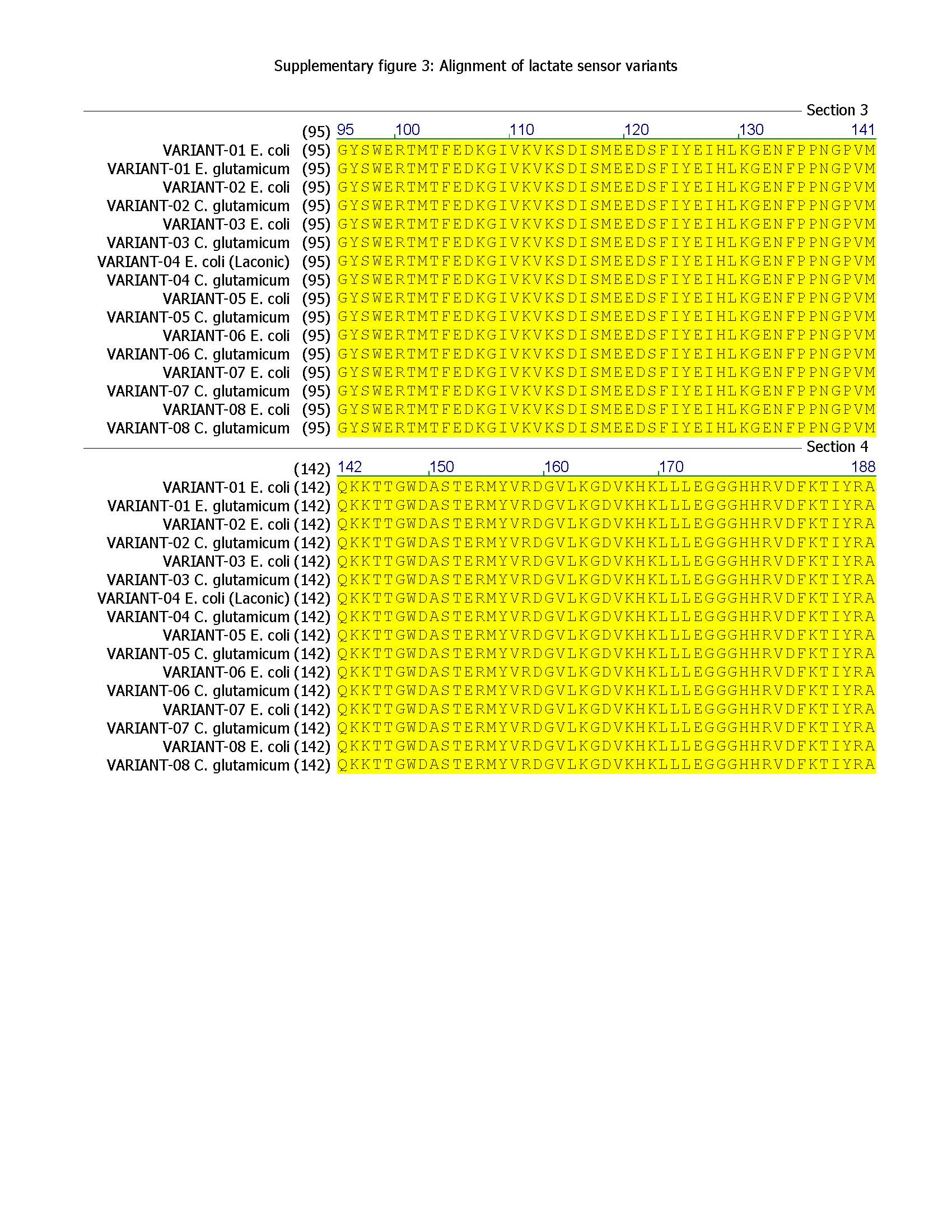


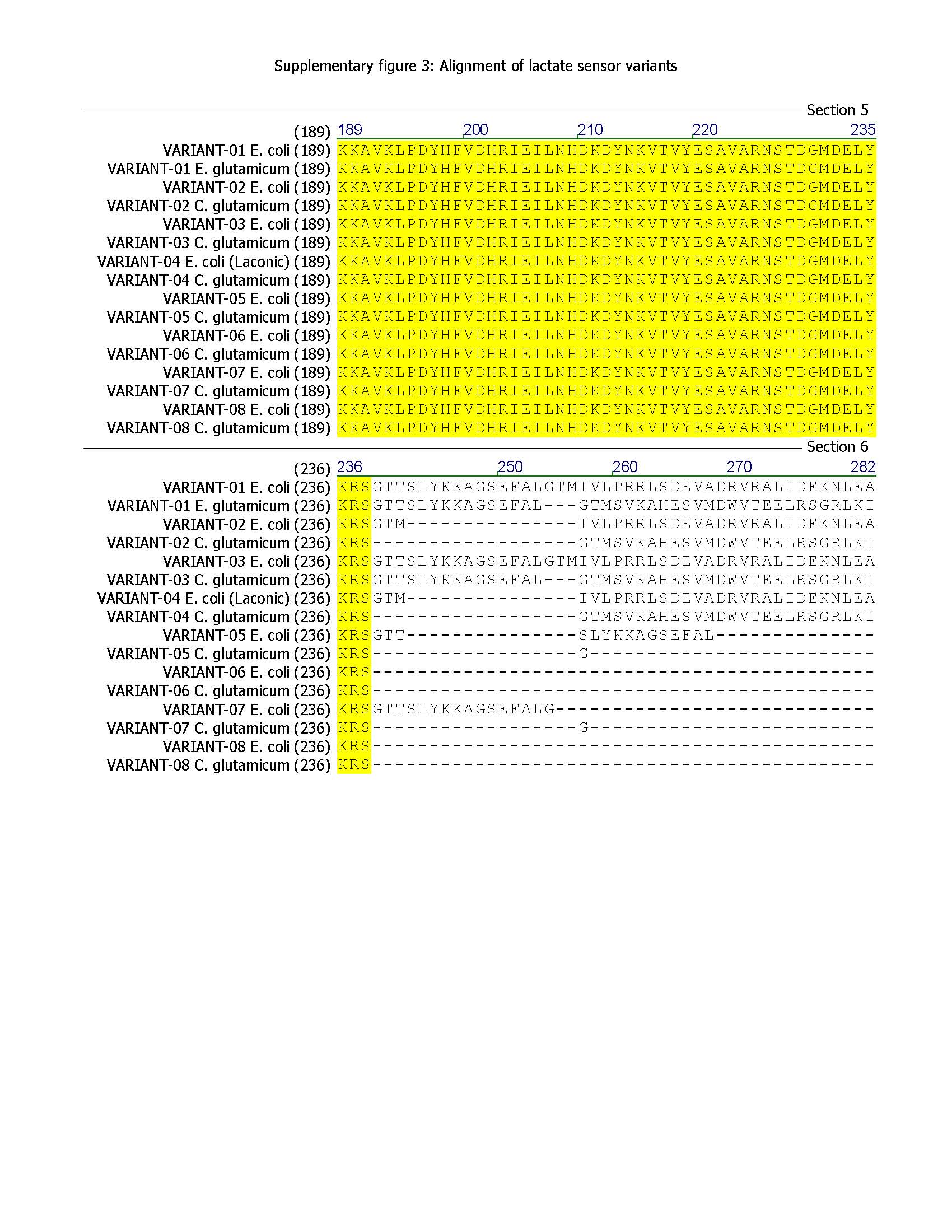


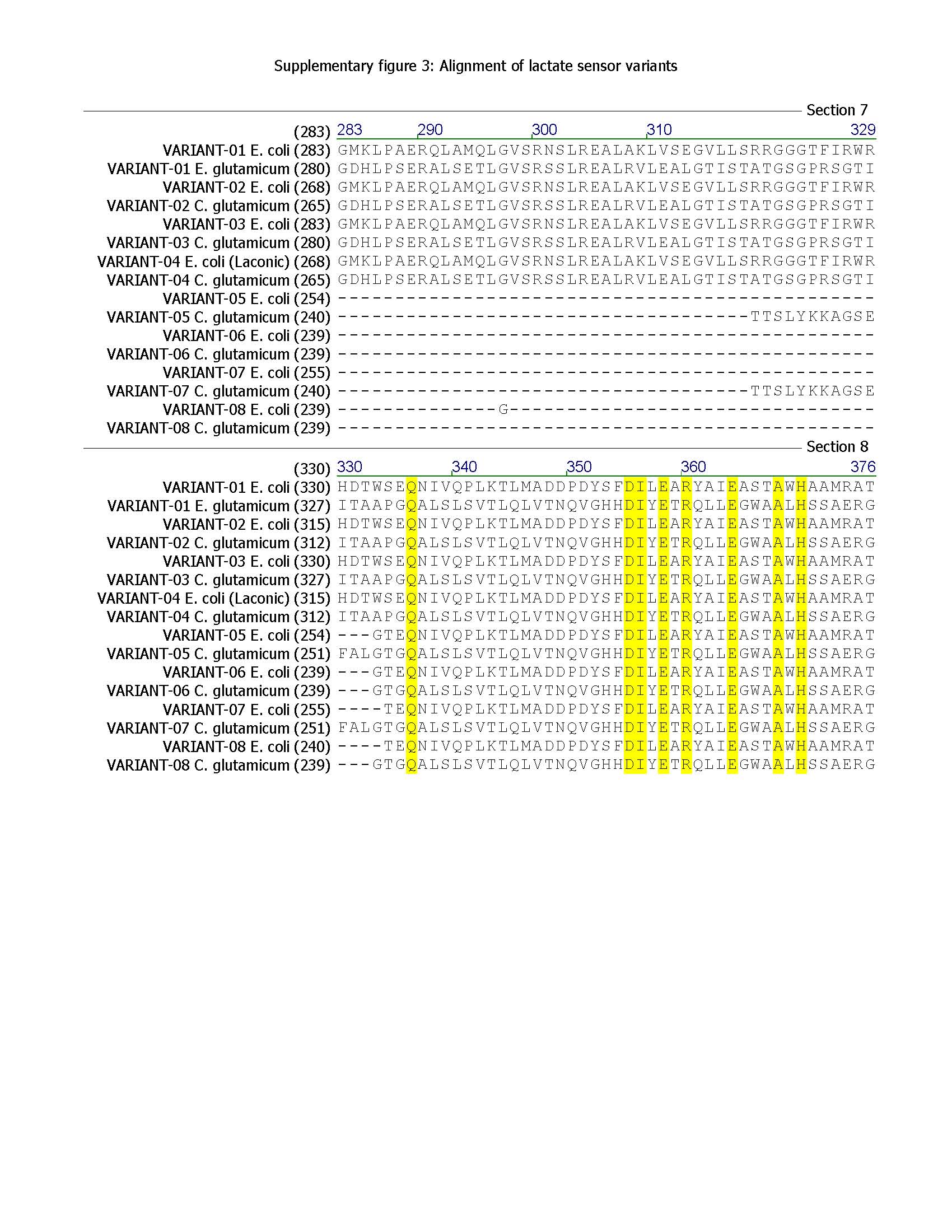


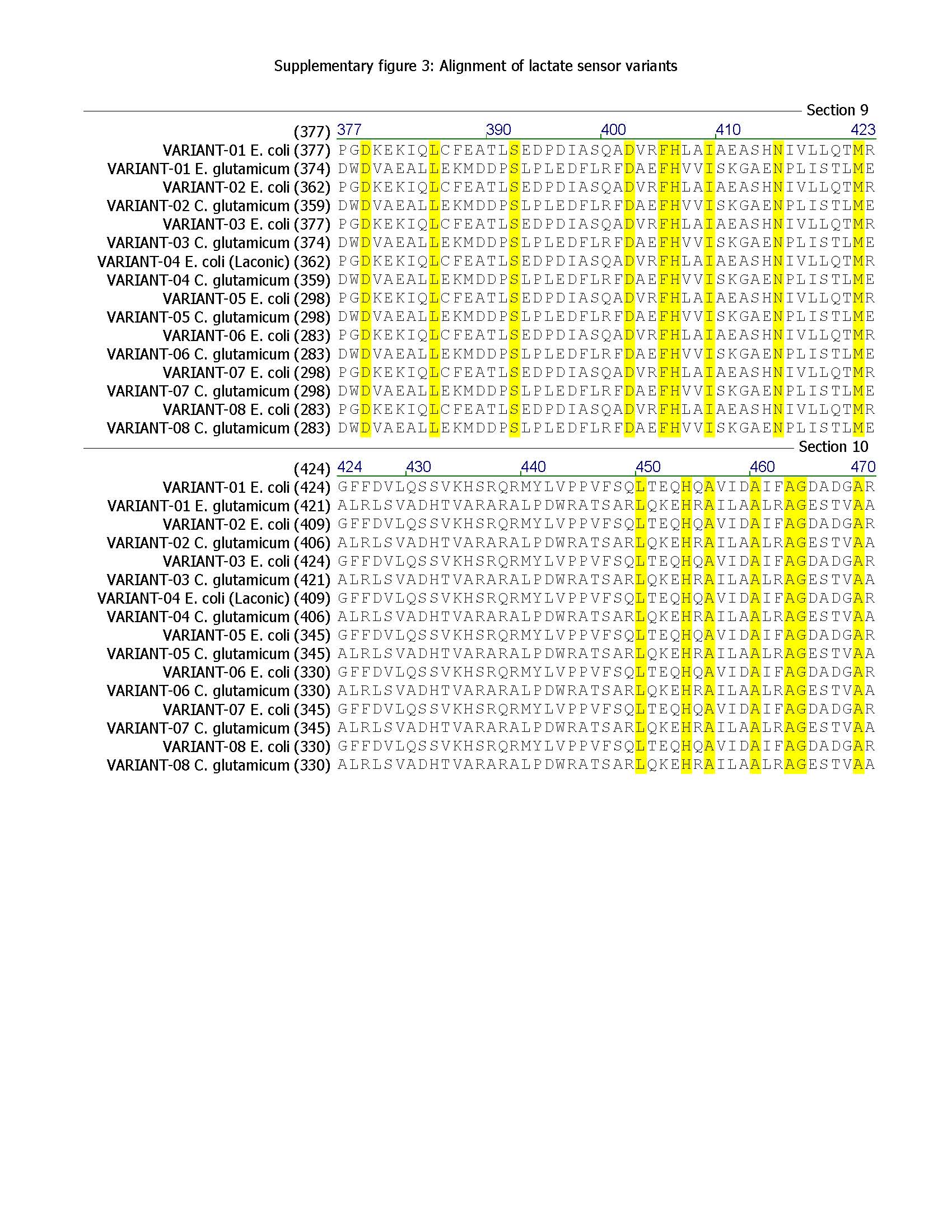


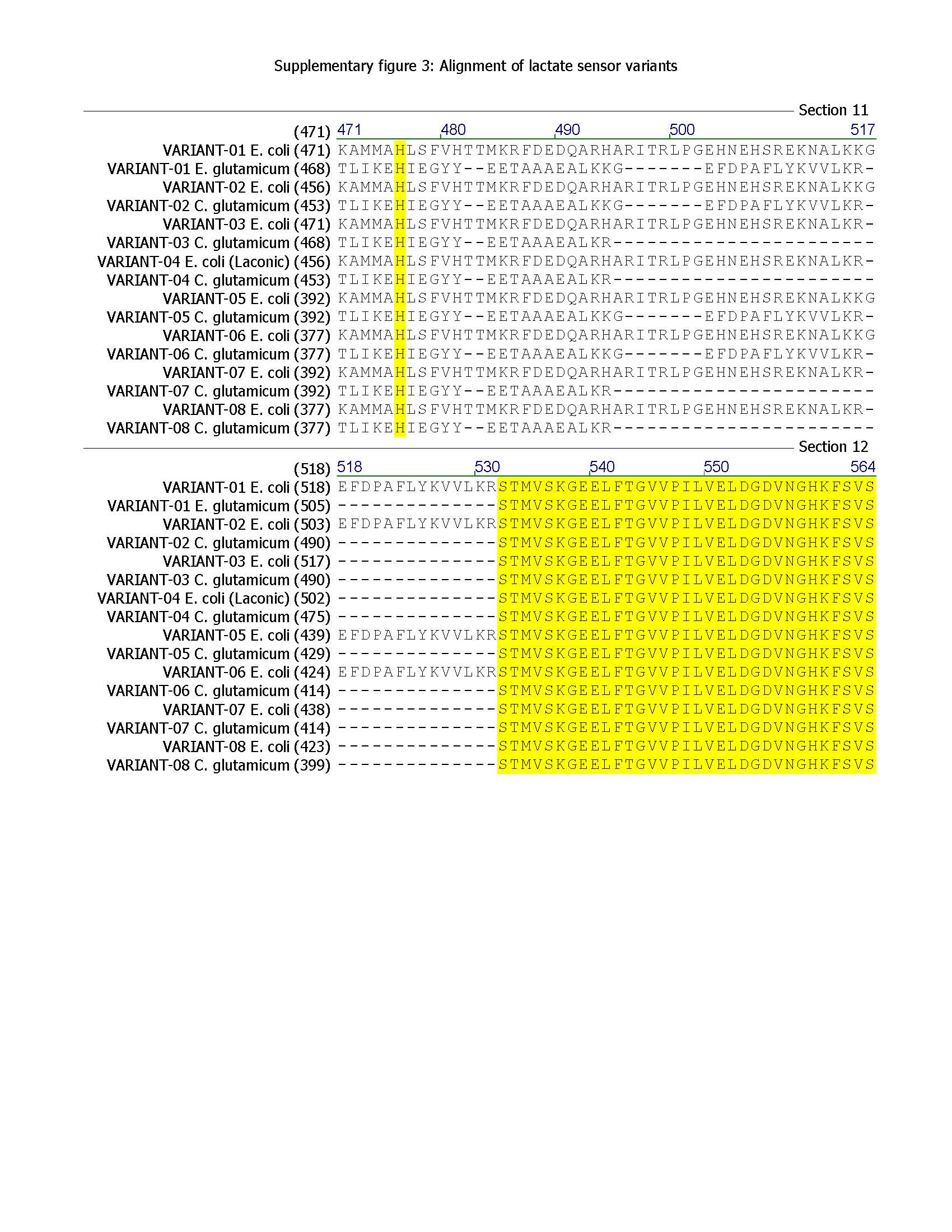


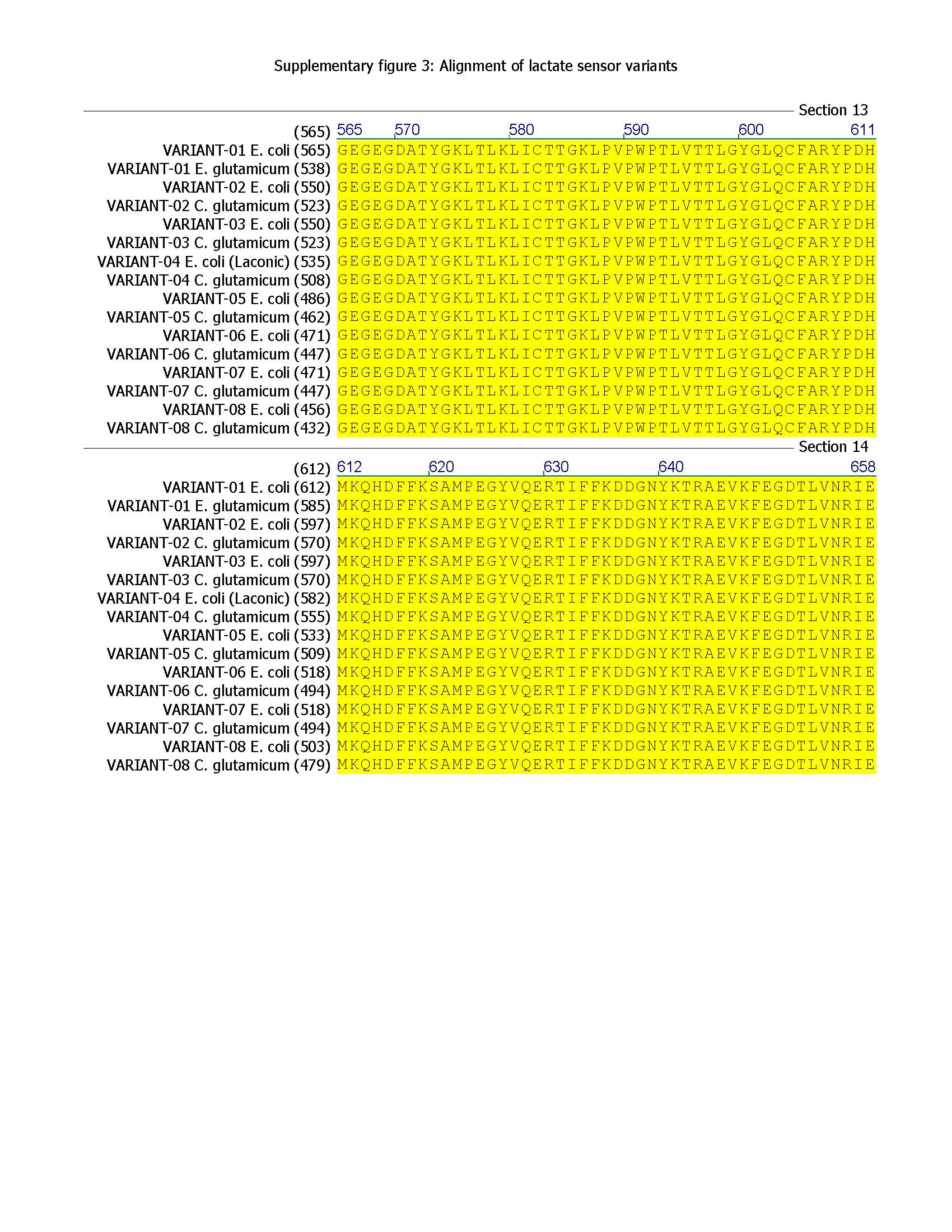

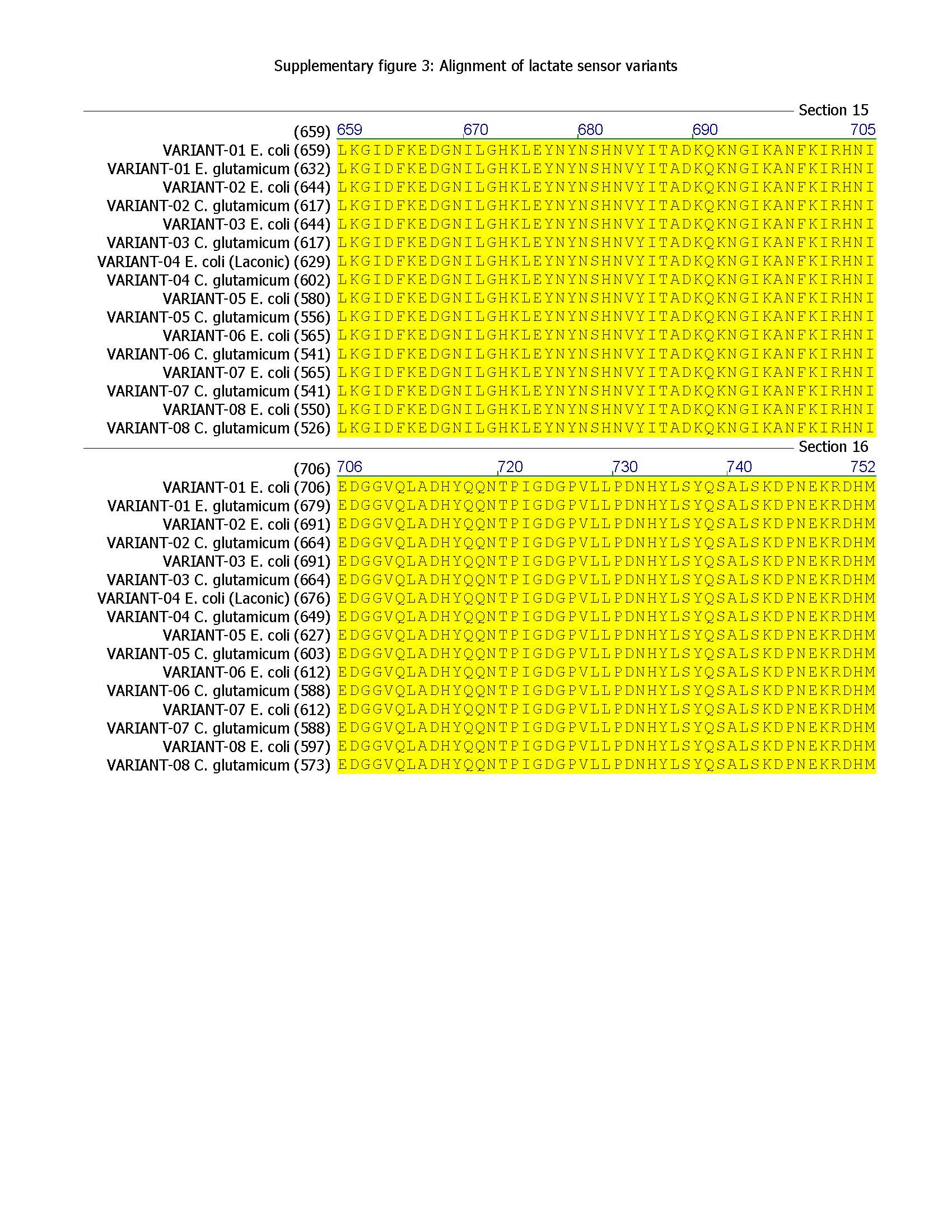


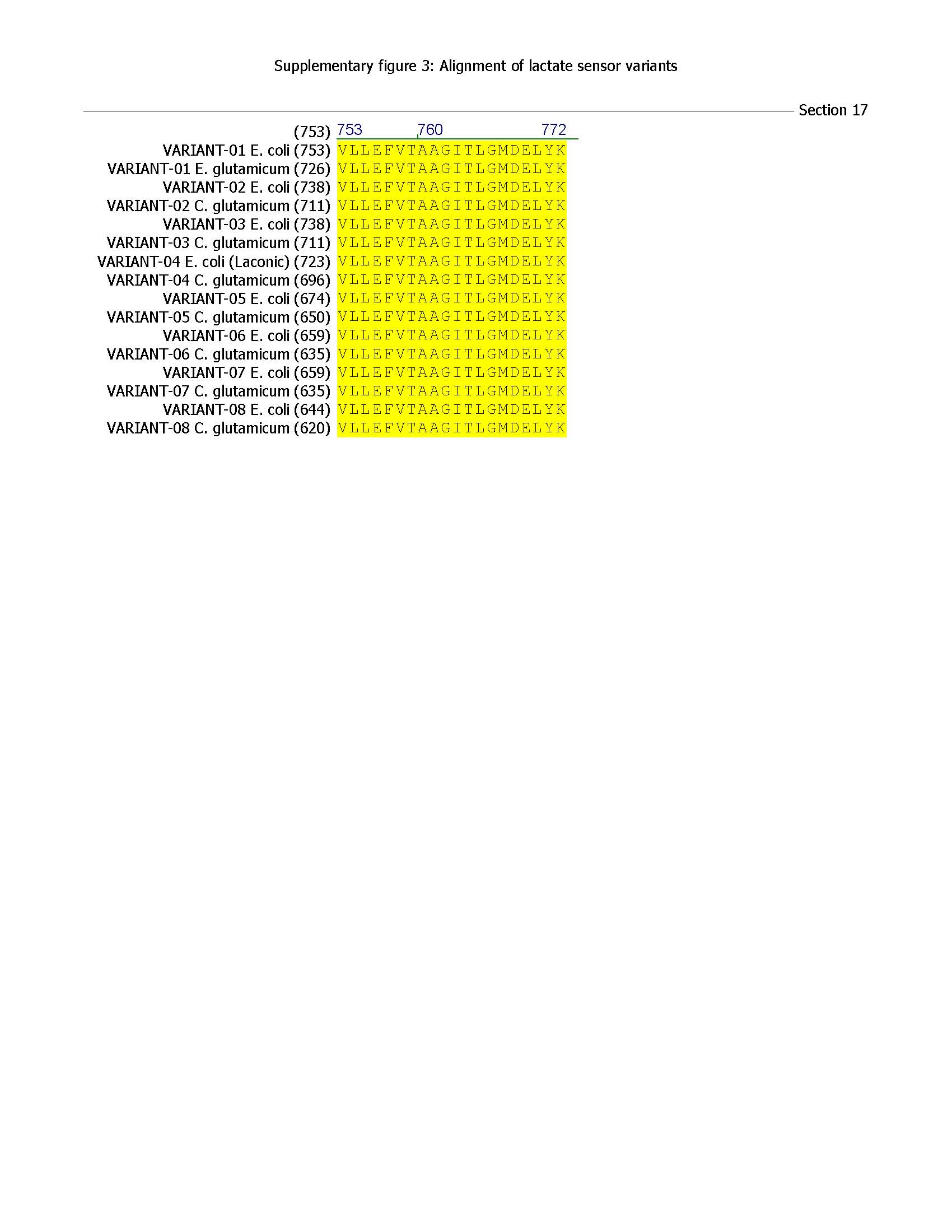


**Figure S1, related to Fig. 1. Alignment of lactate sensor sequences.** Eight variants of the lactate sensor were generated with either LldR from *E. coli* and *C. glutamicum* as described in Experimental Procedures. Variant 04 from *E. Coli* was termed Laconic. Identical amino acid residues are highlighted in yellow.
