# Supplementary material for: A Genetically Encoded FRET Lactate Sensor and Its Use To Detect the Warburg Effect in Single Cancer Cells
Source: PLoS One. 2013 Feb 26;8(2):e57712. doi: 10.1371/journal.pone.0057712 (PMC3582500; doi:10.1371/journal.pone.0057712)
Supplement: Figure S2 — Related to Fig. 3 . Depletion of lactate induced by pyruvate and monochloracetate. (A) Astrocytes were successively exposed to 5 mM lactate, 10 mM pyruvate and 50 mM monochloroacetate (MCA). Time lapse data are from 5 cells in a single experiment. The bar graph summarizes the difference between control ratios and in the presence of pyruvate or MCA for 3 separate experiments. (B) Whole cell lactate was estimated with an enzymatic kit in astrocytic cultures exposed to 2 mM glucose/1 mM lactate (control), after 5 min exposure to 50 mM MCA, or after 5 min exposure to 5 mM lactate. Data are from 3–4 separate determinations. *, p < 0.05 respect to control. (DOC) [file pone.0057712.s002.doc]

**Figure S2. Depletion of lactate induced by pyruvate and monochloracetate**

**Figure S2, related to Fig. 3. Depletion of lactate induced by pyruvate and monochloracetate.** (A) Astrocytes were successively exposed to 5 mM lactate, 10 mM pyruvate and 50 mM monochloroacetate (MCA). Time lapse data are from 5 cells in a single experiment. The bar graph summarizes the difference between control ratios and in the presence of pyruvate or MCA for 3 separate experiments. (B) Whole cell lactate was estimated with an enzymatic kit in astrocytic cultures exposed to 2 mM glucose/1 mM lactate (control), after 5 min exposure to 50 mM MCA, or after 5 min exposure to 5 mM lactate. Data are from 3-4 separate determinations. *, p < 0.05 respect to control.
