# Supplementary material for: A Genetically Encoded FRET Lactate Sensor and Its Use To Detect the Warburg Effect in Single Cancer Cells
Source: PLoS One. 2013 Feb 26;8(2):e57712. doi: 10.1371/journal.pone.0057712 (PMC3582500; doi:10.1371/journal.pone.0057712)
Supplement: Figure S3 — Related to Fig. 4 . Inhibition of lactate uptake by phloretin and pCMBS. T98G glioma cells were exposed to 5 mM lactate in the absence and presence of 50 µM phloretin or 500 µM pCMBS. The straight lines represent the initial slopes of lactate uptake. The bar graphs summarize data for 3 experiments in each cell type. *, p < 0.05 with respect to phloretin. (DOC) [file pone.0057712.s003.doc]

**Figure S3. Inhibition of lactate uptake by phloretin and pCMBS**

**Figure S3, related to Fig. 4. Inhibition of lactate uptake by phloretin and pCMBS.** T98G glioma cells were exposed to 5 mM lactate in the absence and presence of 50 μM phloretin or 500 μM pCMBS. The straight lines represent the initial slopes of lactate uptake. The bar graphs summarize data for 3 experiments in each cell type. *, p < 0.05 with respect to phloretin.
