# Supplementary material for: A Genetically Encoded FRET Lactate Sensor and Its Use To Detect the Warburg Effect in Single Cancer Cells
Source: PLoS One. 2013 Feb 26;8(2):e57712. doi: 10.1371/journal.pone.0057712 (PMC3582500; doi:10.1371/journal.pone.0057712)
Supplement: Figure S4 — Related to Fig. 4 . Effects of azide, phloretin and pCMBS on pH. Intracellular pH was measured with BCECF in astrocytes exposed to 5 mM azide, 50 µM phloretin or 500 µM pCMBS. A control alkalinization with 10 mM ammonium chloride is also shown. Data are from 8 cells in a single experiment. Arrows show the times selected for the bar graphs below, which summarize the fall in intracellular pH for 3 experiments in each cell type. Note that lactate accumulation measurements in Fig. 5 were done at 30 s for azide, at 2 min for phloretin and at 4 min for pCMBS. (DOC) [file pone.0057712.s004.doc]

**Figure S4. Effects of azide, phloretin and pCMBS on pH**

**Figure S4, related to Fig. 4. Effects of azide, phloretin and pCMBS on pH.** Intracellular pH was measured with BCECF in astrocytes exposed to 5 mM azide, 50 μM phloretin or 500 μM pCMBS. A control alkalinization with 10 mM ammonium chloride is also shown. Data are from 8 cells in a single experiment. Arrows show the times selected for the bar graphs below, which summarize the fall in intracellular pH for 3 experiments in each cell type. Note that lactate accumulation measurements in **Fig. 5** were done at 30 s for azide, at 2 min for phloretin and at 4 min for pCMBS.
