# Supplementary material for: A Genetically Encoded FRET Lactate Sensor and Its Use To Detect the Warburg Effect in Single Cancer Cells
Source: PLoS One. 2013 Feb 26;8(2):e57712. doi: 10.1371/journal.pone.0057712 (PMC3582500; doi:10.1371/journal.pone.0057712)
Supplement: Figure S5 — Related to Fig. 5 . Intracellular glucose depletion by azide. The effect of 5 mM azide on intracellular glucose was measured with a FRET glucose sensor (Bitner et al., 2010). The bar graph represents the degree of glucose depletion after 2 min of exposure to azide (arrow) in 3 experiments in each cell type. *, p < 0.05 respect to astrocytes and HEK293 cells. (DOC) [file pone.0057712.s005.doc]

**Figure S5. Intracellular glucose depletion by azide**

**Figure S5, related to Fig. 5. Intracellular glucose depletion by azide.** The effect of 5 mM azide on intracellular glucose was measured with a FRET glucose sensor (Bitner et al., 2010). The bar graph represents the degree of glucose depletion after 2 min of exposure to azide (arrow) in 3 experiments in each cell type. *, p < 0.05 respect to astrocytes and HEK293 cells.
