# Supplementary material for: A Genetically Encoded FRET Lactate Sensor and Its Use To Detect the Warburg Effect in Single Cancer Cells
Source: PLoS One. 2013 Feb 26;8(2):e57712. doi: 10.1371/journal.pone.0057712 (PMC3582500; doi:10.1371/journal.pone.0057712)
Supplement: Figure S6 — related to Fig. 5 . Effect of frequency of acquisition on the estimation of the lactate rise in response to azide. Astrocytes expressing the lactate sensor in the presence of 2 mM glucose were exposed to 5 mM azide while the fluorescence ratio was measured every second (1 Hz). A linear function was fitted to the initial phase of the lactate rise, giving an estimated rate of 0.23 min−1 (left panel). When the data were decimated to simulate a frequency of acquisition of 0.1 Hz, the estimated rate was 0.26 min−1(right panel). The lower graph shows data for 3 different cells. Similar results were obtained in a separate experiment. (DOC) [file pone.0057712.s006.doc]

**Figure S6. Effect of frequency of acquisition on the estimation**

**of the lactate rise in response to azide**

**Figure S6, related to Fig. 5. Effect of frequency of acquisition on the estimation of the lactate rise in response to azide.** Astrocytes expressing the lactate sensor in the presence of 2 mM glucose were exposed to 5 mM azide while the fluorescence ratio was measured every second (1 Hz). A linear function was fitted to the initial phase of the lactate rise, giving an estimated rate of 0.23 min^-1^ (left panel). When the data were decimated to simulate a frequency of acquisition of 0.1 Hz, the estimated rate was 0.26 min^-1^(right panel). The lower graph shows data for 3 different cells. Similar results were obtained in a separate experiment.
