# Supplementary material for: A Genetically Encoded FRET Lactate Sensor and Its Use To Detect the Warburg Effect in Single Cancer Cells
Source: PLoS One. 2013 Feb 26;8(2):e57712. doi: 10.1371/journal.pone.0057712 (PMC3582500; doi:10.1371/journal.pone.0057712)
Supplement: Figure S7 — Related to Fig. 5 . Estimation of the Warburg Index with AR-C155858. (A) The uptake of 5 mM lactate was measured in astrocytes in the absence and presence of 1 µM AR-C155858. Data are from 10 cells in two experiments. (B) An astrocyte expressing Laconic was sequentially exposed to 5 mM azide and 1 µM AR-C155858. The straight lines represent the slopes of lactate accumulation fitted by linear regression within the same range of ratio values. The bar graph shows a summary of the slopes (Δ ratio/min) obtained for 10 cells in two experiments. The calculated Warburg index was 0.07 ± 0.006. (DOC) [file pone.0057712.s007.doc]

**Figure S7. Estimation of the Warburg Index with AR-C155858**

**Figure S7, related to Fig. 5.** **Estimation of the Warburg Index with AR-C155858. (**A**)** The uptake of 5 mM lactate was measured in astrocytes in the absence and presence of 1 μM AR-C155858. Data are from 10 cells in two experiments. (B) An astrocyte expressing Laconic was sequentially exposed to 5 mM azide and 1 μM AR-C155858. The straight lines represent the slopes of lactate accumulation fitted by linear regression within the same range of ratio values. The bar graph shows a summary of the slopes (Δ ratio/min) obtained for 10 cells in two experiments. The calculated Warburg index was 0.07 ± 0.006.
