# Supplementary material for: A Genetically Encoded FRET Lactate Sensor and Its Use To Detect the Warburg Effect in Single Cancer Cells
Source: PLoS One. 2013 Feb 26;8(2):e57712. doi: 10.1371/journal.pone.0057712 (PMC3582500; doi:10.1371/journal.pone.0057712)
Supplement: Figure S8 — Related to Figs. 4 – 5 . Effects of phloretin, AR-C155858 and azide on lactate sensing in vitro. The mTFP/Venus ratio was measured in the stated conditions and expressed relative to the control value in the absence of lactate. Data are from 6 determinations in two different sensor extracts. (DOC) [file pone.0057712.s008.doc]

**Figure S8. Effects of phloretin, AR-C155858 and azide**

**on lactate sensing *in vitro.***

**Figure S8, related to Figs. 4-5. Effects of phloretin, AR-C155858 and azide on lactate sensing *in vitro.*** The mTFP/Venus ratio was measured in the stated conditions and expressed relative to the control value in the absence of lactate. Data are from 6 determinations in two different sensor extracts.
